# Analysis of Secreted Proteins from Prepubertal Ovarian Tissues Exposed In Vitro to Cisplatin and LH

**DOI:** 10.3390/cells11071208

**Published:** 2022-04-03

**Authors:** Serena Marcozzi, Fabiola Ciccosanti, Gian Maria Fimia, Mauro Piacentini, Cinzia Caggiano, Claudio Sette, Massimo De Felici, Francesca Gioia Klinger

**Affiliations:** 1 Section of Histology and Embryology, Department of Biomedicine and Prevention, University of Rome Tor Vergata, 00133 Rome, Italy; sere.marcozzi@gmail.com; 2 Department of Epidemiology, Preclinical Research and Advanced Diagnostics, National Institute for Infectious Diseases ‘L. Spallanzani’ IRCCS, 00149 Rome, Italy; fabiola.ciccosanti@inmi.it (F.C.); gianmaria.fimia@inmi.it (G.M.F.); mauro.piacentini@uniroma2.it (M.P.); 3 Department of Molecular Medicine, Sapienza University of Rome, 00185 Rome, Italy; 4 Department of Biology, University of Rome Tor Vergata, 00133 Rome, Italy; 5 Section of Human Anatomy, Department of Neuroscience, Catholic University of the Sacred Heart, 00168 Rome, Italy; cinzia.caggiano@unicatt.it (C.C.); claudio.sette@unicatt.it (C.S.); 6 GSTEP Organoids Core Facility, IRCCS Fondazione Policlinico Agostino Gemelli, 00168 Rome, Italy; 7 Departmental Faculty, Saint Camillus International University of Health Sciences, 00131 Rome, Italy

**Keywords:** cisplatin, ovary, chemotherapy, microenvironment, LH, ovarian follicles, secretome

## Abstract

It is well known that secreted and exosomal proteins are associated with a broad range of physiological processes involving tissue homeostasis and differentiation. In the present paper, our purpose was to characterize the proteome of the culture medium in which the oocytes within the primordial/primary follicles underwent apoptosis induced by cisplatin (CIS) or were, for the most part, protected by LH against the drug. To this aim, prepubertal ovarian tissues were cultured under control and in the presence of CIS, LH, and CIS + LH. The culture media were harvested after 2, 12, and 24 h from chemotherapeutic drug treatment and analyzed by liquid chromatography–mass spectrometry (LC-MS). We found that apoptotic conditions generated by CIS in the cultured ovarian tissues and/or oocytes are reflected in distinct changes in the extracellular microenvironment in which they were cultured. These changes became evident mainly from 12 h onwards and were characterized by the inhibition or decreased release of a variety of compounds, such as the proteases Htra1 and Prss23, the antioxidants Prdx2 and Hbat1, the metabolic regulators Ldha and Pkm, and regulators of apoptotic pathways such as Tmsb4x. Altogether, these results confirm the biological relevance of the LH action on prepuberal ovaries and provide novel information about the proteins released by the ovarian tissues exposed to CIS and LH in the surrounding microenvironment. These data might represent a valuable resource for future studies aimed to clarify the effects and identify biomarkers of these compounds’ action on the developing ovary.

## 1. Introduction

Premature ovarian failure and female infertility are frequent side effects of anticancer therapies, owing to the extreme sensitivity of the ovarian oocytes’ reserve to the damaging effects of irradiation and chemotherapy on DNA. Alongside the cryopreservation of gametes, several approaches have also been explored with the aim of protecting ovarian follicles and the oocytes they contain from chemotherapy-induced damage (for a review, see Spears et al., 2019 [1]).

In previous papers, we reported a significant and unexpected protective effect of luteinizing hormone (LH) against the cisplatin (CIS)-induced apoptosis of the primordial follicle pool in prepubertal ovaries [2]. Moreover, we confirmed such LH protection both in prepubertal and adult animals [2,3]. In the in vitro system, we also found that LH treatment of the ovarian tissues generated molecular signals in the ovarian somatic cells that, through the activation of PI3K/AKT and cAMP/PKA pathways in the oocytes, prevented their apoptosis [2]. Such signals did not impinge on the activation of TAp63 protein, a major event that, evoked by CIS in oocytes, leads to their apoptosis [2,4,5], but they improved the oocyte capability to repair the DNA damage caused by the drug and counteracted apoptotic pathways [2]. Although some of the LH-dependent signals protecting oocytes from CIS-dependent apoptosis are probably passed from pregranulosa cells to the oocytes through gap junctions, other molecules released in the medium might act both on the pregranulosa cells themselves and oocytes [2,6]. In this regard, secreted and shed (through exosomes) proteins from the ovarian cells may represent components of the microenvironments favoring or preventing the activation of the apoptotic pathways induced by CIS and/or LH both in the pregranulosa cells and oocytes.

In the present study, we collected conditioned media from prepubertal ovarian tissues cultured under control and three different conditions, namely in the presence of CIS, LH, and CIS + LH for 2, 12, and 24 h. Proteins in the conditioned media were analyzed by LC-MS, and the relative abundance of the identified proteins was determined by spectral counting. In this way, information about the most abundant secreted proteins produced by cells of prepubertal ovaries throughout the course of the culture stimulated by LH or exposed to CIS without or with LH was obtained. This approach allowed us to discover novel processes such as those involving specific metalloproteinases and reactive oxygen species (ROS) that might play an important role in modulating the survival and apoptosis of the ovarian somatic cells and oocytes.

## 2. Materials and Methods

### 2.1. Animals

Transgenic p18 GFP/c-Kit [7] mice were housed and mated under standard laboratory conditions in an environmentally controlled room and treated using humane care in order to inflict the least possible pain. All experiments were approved by the Institutional Animal Care and Use Committee (IACUC) and carried out according to the Italian and European rules (D.L.116/92; C.E. 609/86; European Directive 2010/63/EU); authorization n° 391/2016-PR.

### 2.2. Ovarian Fragments Culture

Ovaries were collected from 4dpp transgenic p-18 GFP/c-kit mice as previously reported [2]. Briefly, each ovary was cleaned and fragmented into 8 pieces. Fragments were then plated at 37 °C, and 5% CO_2_ in αMEM (Aurogene, Rome, Italy) supplemented with 10% FBS (Gibco, Milan, Italy), l-glutamine, penicillin-G, and streptomycin, pyruvic acid, *N*-acetyl-l-cysteine, and ITS liquid media supplement (all from Sigma-Aldrich, Milan, Italy). After 4 days in culture, the ovarian fragments formed a thin layer of tissue containing GFP-positive oocytes visible under a fluorescence microscope. At this time (T0 time point), 10 μM CIS or 200 mIU/mL LH was added to the culture medium. For the co-treatment with LH and chemotherapeutic agent, cells were preincubated with LH 1 h before the addition of the drug since we already know it to be effective from our published results [2]. After 2, 12, and 24 h of chemotherapeutic drug treatment, fragments of culture media (FCM) were harvested and frozen at −80 °C until use.

Before removing FCM, we confirmed the effects caused by the treatments on the ovarian tissues by counting the number of the primordial follicle-enclosed oocytes (POs) under an inverted fluorescence microscope under a Leica CTR 6000 microscope, as previously reported [2]. As expected, no evident differences were observed in LH, CIS, and CIS + LH groups with respect to the CTRL after 2 and 12 h in culture (data not shown), whereas, after 24 h, LH’s protective effect against CIS-induced apoptosis of POs was confirmed (Figure S1).

### 2.3. LC-MS/MS Analysis

Proteins in the FCM were precipitated with ethanol 100% and resuspended in lysing buffer (Dynabeads Co-Immunoprecipitation Kit, ThermoFisher Scientific, Waltham, MA, USA, 14321D) complemented with protease and phosphatase inhibitors (Protease Inhibitor Cocktail plus (Sigma-Aldrich, P8340), 5 mM sodium fluoride (Sigma-Aldrich, S-7920), 0.5 mM sodium orthovanadate (Sigma-Aldrich, S6508), 1 mM sodium molybdate (Sigma-Aldrich, S-6646). and 0.5 mM phenylmethylsulphonyl fluoride (Sigma-Aldrich, P7626)). The protein suspension was then boiled at 100 °C for 5 min. Disulfide bonds were reduced and alkylated, respectively, with 10 mM dithiothreitol at 56 °C for 30 min and 55 mM iodoacetamide at RT for 20 min. After precipitation with ethanol 100%, the samples were resuspended in 40 μL NH_4_HCO_3_ 50 mM, 2 M urea, and digested by trypsin 0.2 μg at 37 °C overnight. Then peptides were purified through a C18 microcolumn ZipTip (Millipore, Milan, Italy, ZTC18S096), eluted from the C18 bed with 10 μL of an ACN 80%/TFA 0.1% solution, dried in a speedvaac and resuspended with 10 μL of ACN 2.5%/TFA 0.1% and 10 μL formic acid.

The peptide mixtures were analyzed by ultra-high-performance liquid chromatography coupled with high-resolution mass spectrometry using a Thermo Scientific Q Exactive Plus Orbitrap; in particular, the peptides were separated by nano liquid chromatography (UltiMate 3000 RSLC nano-LC system, ThermoFisher Scientific), loaded onto a 75 μm C18 column (ES800-ThermoFisher Scientific), using a 100 min multistep gradient elution (from 4 to 90% eluent B with a constant flow of 0.3 μL/min). They were analyzed by an Exactive Q plus mass spectrometer (ThermoFisher Scientific) [8].

The raw data from the mass spectrometric analysis were processed using the MaxQuant software v.1.5.5.1.

The mouse reference proteome set from UniProt was used to identify peptides and proteins. Protein groups containing matches to proteins from the reversed database or contaminants were discarded. The data file obtained includes peptide and protein identification, accession numbers, protein and gene names, sequence coverage, and intensity-based absolute quantification (iBAQ) values of each sample. Data sets were log_2_-transformed and filtered for proteins with two or more unique peptides and 100% confidence. The mean of log_2_ iBAQ values from three different experiments was calculated for each metabolite in the analysis (Mean log_2_ iBAQ). Heatmap, hierarchical clustering, and PCA analyses of the FCM were performed on the Z-transformed Mean log_2_ iBAQ with the Perseus software v.1.6.12. Violin plots and Venn diagrams were generated using GraphPad Prism v9.0 and Orange Data Mining v3.3 software. To compare differences in the protein amount between samples, the fold change (FC) was calculated as follows: FC sample 1 vs. sample 2 = (Mean log_2_ iBAQ sample 1) − (Mean log_2_ iBAQ sample 2). 

### 2.4. Statistical Analysis

All data represent the mean (±standard deviation, SD) of three independent experiments. Two-tailed Student’s *t*-test calculations were used in statistical tests. A protein was considered statistically significant if its fold change was ≥2 and *p*-value < 0.05. Statistical significance was based on *p*-values: * *p* < 0.05, ** *p* < 0.01, *** *p* < 0.001, **** *p* < 0.0001.

## 3. Results

### 3.1. General Profiles of Proteins in the Culture Media 

The LC-MS analysis of the proteins in the FCM from all groups (CTRL, CIS, LH, and CIS + LH) at 2, 12, and 24 h of culture revealed a total of 340 proteins across samples. Filtering for proteins with two or more unique peptides and a 100% confidence reduced the dataset to 170 different high- and medium-abundant proteins in the media (Data S1 and Table S1). Potentially secreted peptides and proteins (114/170 = 67.06%) were identified using the mouse reference proteome set from UniProt [9]; 56 out of 170 proteins were predicted as intracellular. However, according to the Vesciclepedia database [10,11], 35 of these proteins can be exosomal. Since exosomes were not isolated from our experimental groups to confirm the exosomal origin of proteins, the 35 proteins identified through the Vesciclepedia database were considered “potentially exosomal” and thus included in the analysis. Therefore, the total amount of potentially secreted proteins rose to 148 (148/170 = 87.06%), and only 22/170 (12.94%) could be considered exclusively intracellular. These latter were likely released into the medium following cell lysis and were not considered in our analyses. The complete list of the proteins identified by our analyses is presented in Table S1.

**Figure 1:**
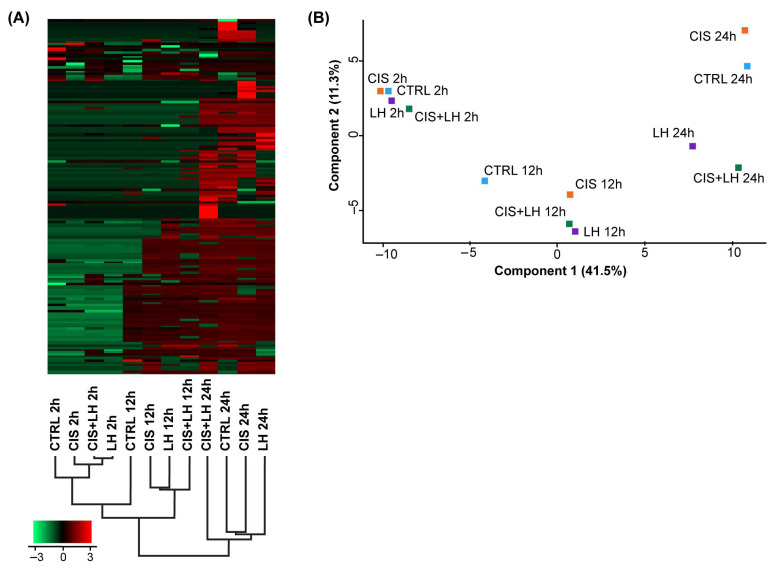
Heatmap combined with hierarchical clustering and principal component analysis (PCA) of FCM after 2, 12, and 24 h of treatment. In (**A**) a heatmap comparison of the 148 secreted metabolites, with two or more unique peptides and a 100% confidence, is depicted. Protein iBAQ values were log_2_-normalized, and cluster analysis was performed using Z-score protein intensities. Red indicates a high expression level; green indicates a low expression level. In (**B**) PCA analysis plot of FCM at 2, 12, and 24 h after treatment.

The heatmap of the Z-transformed Mean log_2_ iBAQ values from the 148 identified proteins showed a very clear and consistent co-segregation of samples in the function of the time of culture, with the major node distinguishing samples of 2 from those of 12 and 24 h (Figure 1A). This pattern was confirmed by principal-component analysis (PCA), in which a distinct separation and aggregation of proteins among different culture times and treatments were clearly observed (Figure 1A).

**Figure 2:**
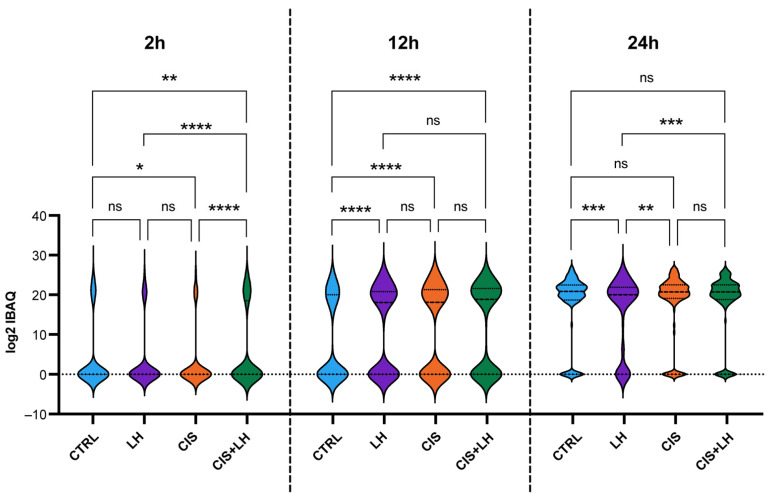
Violin plot representation of the metabolites expressed in each FCM after 2, 12, and 24 h of treatment. Mean log_2_ iBAQ values of the 148 secreted metabolites were considered at each time point in the analysis. Statistically significant differences between indicated groups * *p* < 0.05; ** *p* < 0.01; *** *p* < 0.001; **** *p* < 0.0001.

The Violin Plot distribution showed protein accumulation throughout the culture time; out of the 148 proteins investigated, 45 were expressed after 2 h, 102 after 12 h, and 147 after 24 h of culture (Figure 2). The time course of the protein accumulation in the FCM varied among the different experimental conditions. Media from the CIS + LH group showed a higher number of proteins at 2 h in comparison to the others (CTRL = 29; LH = 25; CIS = 23; CIS + LH = 41); at 12 h, media from the LH, CIS, and CIS + LH groups contained a similar number of proteins, which was higher than CTRL (CTRL = 62; LH = 85; CIS = 92; CIS + LH = 88); at 24 h, in media from LH group, the number of proteins was slightly lower in comparison to the others (CTRL = 126; LH = 117; CIS = 128; CIS + LH = 128) (Figure 2).

**Figure 3:**
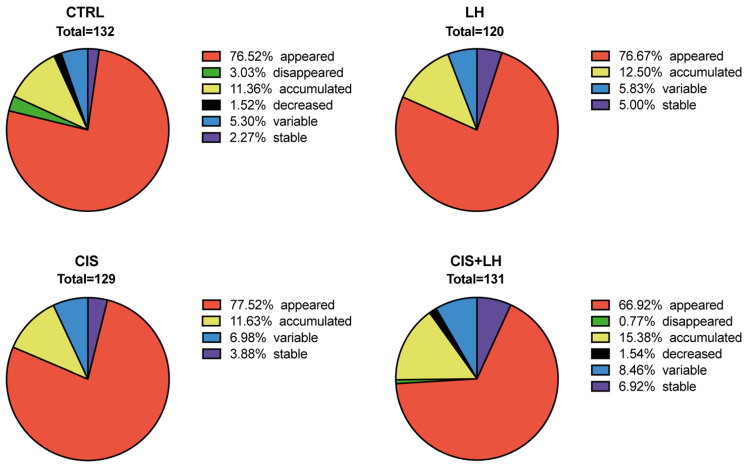
Metabolites’ percentage trend during culture time in CTRL, LH, CIS, and CIS + LH. Appeared: proteins that were not expressed at 2 h but appeared during culture time (red). Disappeared: proteins whose expression disappeared during the culture period (green). Accumulated: proteins already expressed at 2 h time point and whose expression increased during the culture time (yellow). Decreased: proteins expressed at 2 h of culture and whose expression decreased during the 24 h of analysis (black). Variable: proteins with a variable expression mode over time (blue). Stable: proteins whose expression did not change during the period of culture analyzed (purple).

The analysis of the proteome variation during time revealed that, depending on groups, about 20–30% of the proteins were already present at 2 h of culture and accumulated (CTRL = 15; LH = 15; CIS = 15; CIS + LH = 20), decreased (CTRL = 2; LH = 0; CIS = 0; CIS + LH = 2), disappeared (CTRL = 4; LH = 0; CIS = 0; CIS + LH = 1), remained stable (CTRL = 3; LH = 6; CIS = 5; CIS + LH = 9), or showed a variable pattern (CTRL = 7; LH = 7; CIS = 9; CIS + LH = 11), throughout the culture. On the other hand, the majority of proteins (from about 70 to 80%) appeared during culture (CTRL = 101; LH = 92; CIS = 100; CIS + LH = 87) (Figure 3 and Table S2).

**Figure 4:**
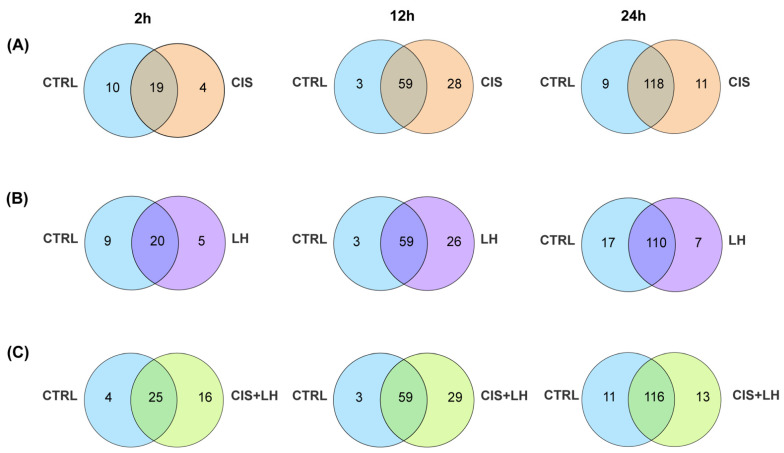
Venn diagrams showing unique and shared proteins between CTRL and the other experimental conditions. The numbers indicate the metabolites counts in the indicated area. (**A**) Overlaps between CTRL and CIS at 2, 12, and 24 h of treatment. (**B**) Overlaps between CTRL and LH at 2, 12, and 24 h of treatment. (**C**) Overlaps between CTRL and CIS + LH at 2, 12, and 24 h of treatment.

The Venn diagrams in Figure 4A–C show that at 2 h, the CTRL samples shared 19, 20, 25 out of 29 proteins, at 12 h, 59, 59, and 59 out of 62 proteins and, at 24 h, 118, 110, and 116 out of 127 proteins with CIS, LH, and CIS + LH groups, respectively. These results indicated that relatively few proteins distinguished CTRL from the treated samples at each time. Specifically, 4, 28, and 11 proteins (total 43) were present at 2, 12, and 24 h, respectively, in the group exposed to CIS and not in the CTRL (Figure 4A). At these culture times, 5, 26, and 7 proteins (total 38, Figure 4B) and 16, 29, and 14 (total 59, Figure 4C) distinguished LH and CIS + LH groups from CTRL, respectively. Of note, the highest number of differentially expressed proteins (DEPs) in the comparisons between CTRL and the treated groups was constantly observed at 12 h.

**Figure 5:**
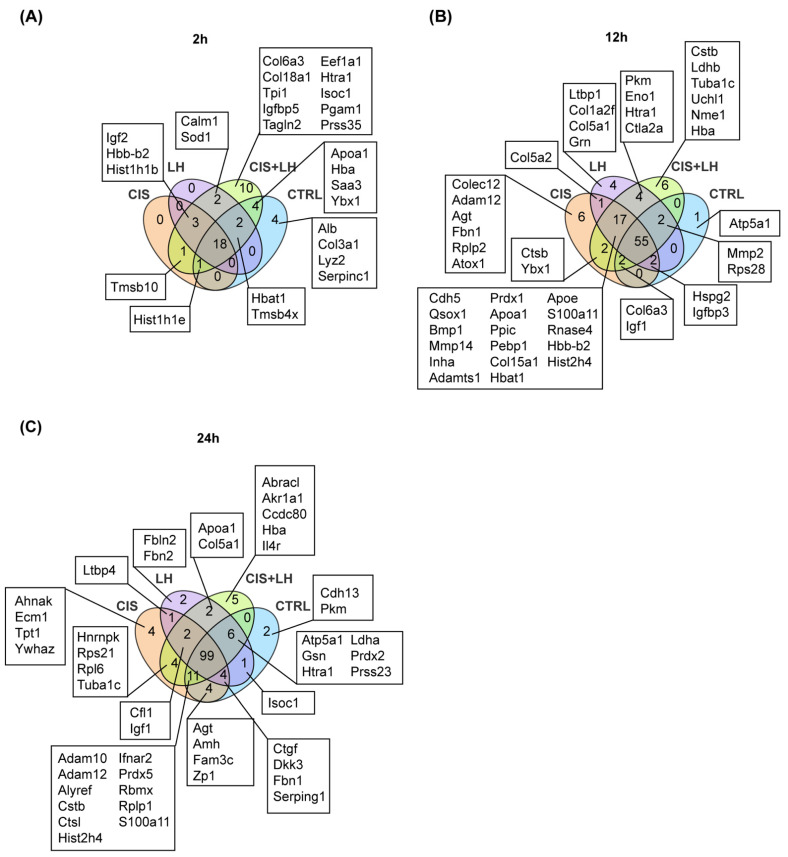
Venn diagram showing unique and shared proteins between all experimental conditions. The numbers indicate the metabolites counts in the indicated area. The metabolites are indicated below the diagram for each category, except for those shared among all groups (reported in Table S3). (**A**) Overlaps between CTRL, LH, CIS, and CIS + LH at 2 h of treatment. (**B**) Overlaps between CTRL, LH, CIS, and CIS + LH at 12 h of treatment. (**C**) Overlaps between CTRL, LH, CIS, and CIS + LH at 24 h of treatment.

**Figure 6:**
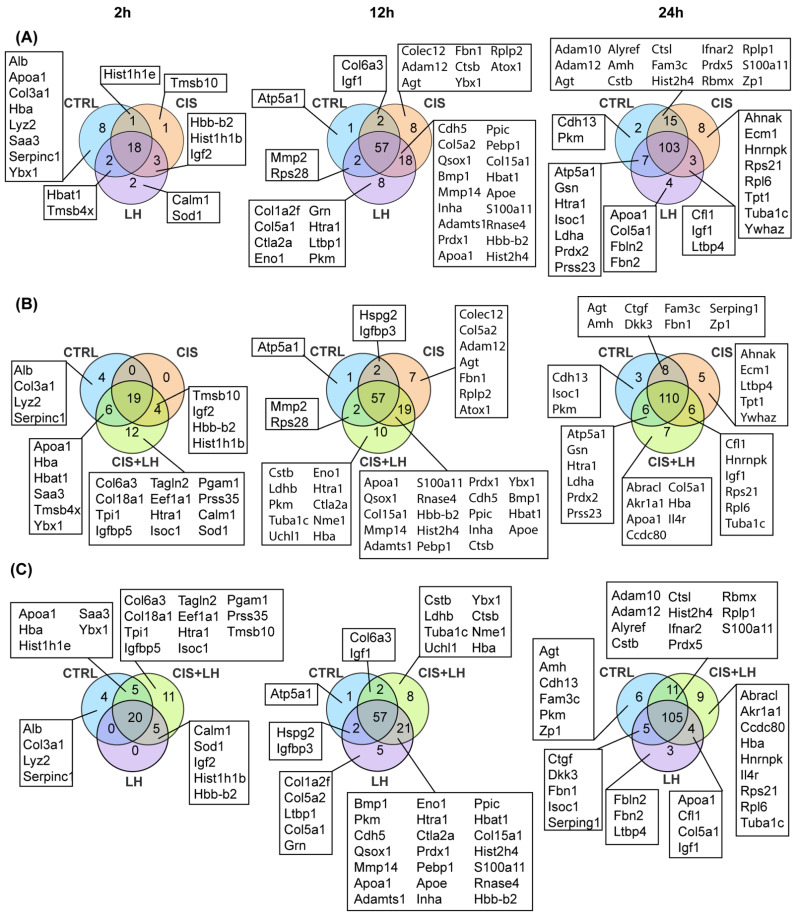
Venn diagram showing unique and shared proteins between indicated groups. The numbers indicate the metabolites counts in the indicated area. The metabolites are indicated below the diagram for each category, except for those shared among groups considered (reported in Table S3). (**A**) Overlaps between CTRL, LH, and CIS at 2, 12, and 24 h of treatment. (**B**) Overlaps between CTRL, CIS, and CIS + LH at 2, 12, and 24 h of treatment. (**C**) Overlaps between CTRL, LH, and CIS + LH at 2, 12, and 24 h of treatment.

By comparing the proteome among all groups and the different experimental conditions (Figure 5 and Table S3), we observed that an increasing number of proteins were shared between CTRL and other groups: 18 out of 45 proteins (40.00%) after 2 h, 55 out of 102 (53.92%) after 12 h and 99 out of 147 (67.35%) at the end of the culture period. The greatest differences in number were detected in the comparisons between CIS + LH vs. LH (16 proteins at 2 h, 10 at 12 h, and 20 at 24 h) and CIS + LH vs. CIS (18 at 2 h, 12 at 12 h, and 13 at 24 h), while minor differences were detected in the comparison between LH vs. CIS + LH (0 at 2 h, 7 at 12, h and 8 at 24 h) (Figure 6 and Figure S2a–c). These results indicated that the presence of both rather than single compounds induced greater numbers of proteins and that LH exerted a prominent role in comparison to CIS when the compounds were used together.

### 3.2. Proteosome Comparison at 2 h

A few differences were detected in the analyses carried out in the FCM from all groups at 2 h (Figure 5 and Figure 6). Likewise, statistical analyses performed on 18 potentially secreted proteins common to FCM of all groups or those shared by paired groups, revealed only a few differences related to the higher level of secreted Cst3 in CIS + LH in comparison to CTRL and CIS and of Dag1 and Anti-dectin-1 15E2 heavy chain in CIS + LH compared to LH (Figure S3). 

No proteins were exclusive of CIS or LH, whereas potentially secreted Alb, Col3a1, Lyz2, Serpinc1 were present only in CTRL, and potentially secreted Col6a3, Col18a1, Htra1, Prss35, Igfbp5, Eef1a1, Isoc1, Tagln2, Tpi1, Pgam1 proteins were found exclusively in CIS + LH. Except for Htra1 and Isoc1, two serine proteases involved in DNA repair [12,13] that were never found in CIS alone, all other proteins appeared or disappeared with different kinetics in the other groups at later times (see also below). 

Potentially secreted Apoa1 and Saa3, both involved in lipid metabolisms, Hba (hemoglobin subunit alfa) and Ybx1, were found in CTRL and CIS + LH only. Apoa1 and Hba disappeared from CTRL throughout the culture while they remained substantially stable in CIS + LH; Ybx1 varied in CTRL and was stably present in CIS + LH; conversely, Saa3 accumulated in CTRL and varied in CIS + LH. From 12 h onwards, Apoa1, Saa3, Ybx1 also appeared in LH and variably in CIS, while Hba was never found in LH or CIS. 

Calm1, a member of the calmodulin family, and Sod1, a superoxide dismutase, were found only in CIS + LH and LH and accumulated thereafter, while they appeared in CTRL and CIS only a 12 h, suggesting an early induction by the hormone. Conversely, Tmsb10, a protein exerting an important role in the organization of the cytoskeleton and participating in apoptosis [14,15,16,17,18], was present in CIS + LH and CIS, accumulated in both groups throughout the culture and appeared in CTRL and LH at 12 h and 24 h, respectively, thus, implying a possible early induction by CIS and no modulation by LH. Hist1h1b, a histone probably mediating exosomal uptake [19], and two potentially secreted proteins Igf2, structurally similar to insulin, and Hbb-b2, a hemoglobin beta-2 subunit, were present in CIS + LH, LH, and CIS but not in CTRL. Hbb-b2 remained stable in CIS + LH, variable in CIS or LH at later culture times, and appeared in CTRL at 24 h; Igf2 and Hist1h1b appeared at 12 h in CTRL and thereafter remained basically stable in all groups, suggesting, for all these proteins, functions mostly independent from the presence of the drug and hormone. 

Finally, Hist1h1e, another histone mediating exosomal uptake (see above), was detected in CTRL, CIS, and CIS + LH but not in LH alone; it remained stable in CTRL and CIS + LH, variable in CIS, and appeared at 12 h in LH, implying an initial inhibitory action of the hormone not maintained in the presence of CIS. Secreted Hbat1, an isotype of hemoglobin alfa with a potential role as an antioxidant molecule in ovarian follicles [20,21], and Tmsb4x, a member of the thymosin family with a role in preventing apoptosis in certain cell types [22,23], were present in CTRL, LH, and CIS + LH but not in CIS, suggesting inhibition by CIS removed by LH; their presence was, however, variable in all groups at a later time.

### 3.3. Proteosome Comparison at 12 h

At this time, only 5 out of 55 potentially secreted proteins shared by all groups were present in statistically different amounts, Lyz2 and Saa3 in CIS vs. CTRL, Anti-dectin-1 15E2 heavy chain in CTRL vs. LH, Dag1 and Psap in CIS + LH vs. CTRL, and Psap in CIS + LH vs. LH (Figure 5 and Figure S2). When common proteins in paired groups were compared, only Col5a2 presented in higher amounts in CIS vs. LH (Figure S3). 

Only one potentially secreted (Atp5a1) protein appeared exclusively in CTRL, while six new proteins were detected only in CIS (secreted Colec12, Adam12, Agt, Fbn1, Atox1, and Rplp2), four in LH (secreted Ltbp1, Col1a2f, Grn, Col5a1), and six in CIS + LH (Cstb, Tuba1c, Uchl1, Nme1 and Ldhp). Atp5a1, a protein of the ATP synthase complex that in soluble form may play a role in regulating gene expression and alternative splicing [24], subsequentially appeared at 24 h in LH and CIS + LH but not in CIS, implying an inhibitory action by the drug removed by LH. Secreted Colec12, a scavenger receptor, Atox1, a copper metal-chaperone protein, and the ribosomal protein Rplp2, found initially only in CIS, appeared in all other groups at 24 h, entailing initial induction by the drug but an unimportant role in the maintenance of the cultured ovarian tissues. The other secreted proteins showed different courses: at 24 h, metalloprotease Adam12 also appeared in CTRL and CIS + LH, but not in LH; the member of the ovalbumin–serine proteases inhibitor family Agt also appeared in CTRL; the large ECM protein Fbn1 was found in CTRL and LH but missed in CIS + LH. The possible meaning of such differential expression will be discussed below. 

Among proteins only in LH at 12 h, Ltbp1, belonging to the family of TGF-β binding proteins, collagenic Colla2f, and Grn, an endoplasmic reticulum stress-responsive factor, appeared in the other groups at 24 h, denoting a relatively early induction by LH, delayed by CIS, and also occurring over time in CTRL and CIS + LH. Moreover, the collagenic Col5a1 protein appeared at 24 h in CIS + LH but remained absent in CTRL and CIS. Since Ltbp1, Grn, and Col5a1 have been recently been shown to favor cell survival by inhibiting various apoptotic pathways [25,26,27], we can postulate a rapid inductive effect of LH on these proteins but an effective antiapoptotic action against CIS only for Col5a1, how we will discuss below. Concerning the proteins present only in CIS + LH, the potentially secreted Ldhb, Nme1, and Uchll appeared thereafter in all other groups. Furthermore, CstB, a cathepsin inhibitor, appeared in CTRL and CIS but not in LH at 24 h, whereas Tuba1c, a tubulin β-isotype, was also found at 24 h in CIS but not in CTRL and LH. From such a complex course, one might infer that CIS favors the secretion of Cstb and Tuba1c, whereas LH exerted the opposite effect overcome by the drug. Intriguingly, since Cstb and Tuba1c have been shown to usually inhibit apoptosis [28,29], if they exert such an effect in the present system, this would be effective only in the presence of LH.

Fourteen of the other potentially secreted proteins appearing at 12 h were common to CIS, LH, and CIS + LH and were found in CTRL only at 24 h (Figure 5 and Figure 6), suggesting accelerated induction by either CIS and LH and no mutual interference, as well as denoting no obvious roles in the present culture system. Moreover, collagenic Col5a11 was found in LH and CIS only, at higher amounts in the latter, implying induction by both compounds and reciprocal inhibition; Ctsb, belonging to the peptidase C1 family and usually favoring autophagy and apoptosis, was present only in CIS and CIS + LH, suggesting induction by the drug and no effect of LH. Conversely, Eno1, a glycolytic enzyme with antiapoptotic activity [30], Ctla2a, a selective inhibitor of cathepsin L-like cysteine proteinases highly expressed in germline and hematopoietic stem cells [31,32], and Pkm, a phosphotyrosine-binding protein involved in glycolysis, were found only in LH and CIS + LH, implying an induction by LH possibly favoring the ovarian tissue maintenance, not hindered by CIS. Interestingly, Cst3, another cysteine protease inhibitor, although detected at 2 h in the media of all groups, was present at significantly higher concentrations in LH and CIS + LH.

Other proteins appearing with different combinations and kinetics in the experimental groups were secreted Igfbp3, Col6a3, Igf1, Mmp2, Hspg2, and Rps28. Considering such variable courses, no clear roles for these proteins in the present system can be postulated.

As a note, Zp2 and Zp3 glycoproteins of the zona pellucida secreted specifically by the growing oocytes appeared at 12 h in all groups, where Zp1, the third glycoprotein of the mouse zona, appeared subsequently in CTRL and CIS at 24 h but remained absent in LH and CIS + LH implying an inhibitory effect of the hormone. 

### 3.4. Proteosome Comparison at 24 h

Among the 99 potentially secreted proteins found in the FCM of all groups at this time (Figure 5), statistical analyses showed only a few significant differences. Namely, Rplp2 and Cdh5 were present at higher levels in LH vs. CTRL, the amount of this latter was also higher in CTRL vs. CIS + LH, and CIS vs. either LH or CIS + LH; Apoe and Igfbp7 were higher in CTRL vs. CIS + LH. The latter was also higher in LH vs. CIS + LH and CIS vs. CIS + LH. Calm1 and Col6a1 were more concentrated in CIS vs. LH and Nme1 in CIS + LH vs. CIS (Figure S3). The same applies when comparing common proteins in paired groups in which the only differences were found in Htra1, which was higher in CTRL vs. CIS + LH, and Cfl1 was higher in CIS vs. LH. Some clues about the possible meanings of the presence of these or other members of the same family proteins in the media of the experimental groups have been given above or discussed below. 

Data in Figure 5 and Figure 6 showed that the only potentially secreted protein appearing exclusively in CTRL at 24 h was Cdh13, a GPI-anchored member of the cadherin superfamily, which lacking direct contact with cytoskeleton is not involved in cell–cell adhesion but participates in Ca^2+^-mobilization and changes of cell phenotype [33]. Although Cdh13 expression and function are associated with ovary development and granulosa cell tumors [34], the meaning of its late expression here and the apparent inhibition by CIS and LH is undetermined. 

The presence of secreted Ahnak, Ecm1, Tpt1, and Ywhaz only in CIS might be linked to the ongoing apoptotic processes in the cultured ovarian tissues at this time but in an opposite way. Ahnak, an unusual desmoyokin scaffolding protein characterized by its large size of approximately 700 kDa [35], Ecm1, involved in ECM remodeling [36], and Tpt1, encoded by a p53 target gene, might be released in an attempt to alleviate apoptosis [37,38], whereas Ywhaz, belonging to the 14-3-3 family hub proteins involved in many signal transduction pathways, including apoptosis [39], could favor this process. Moreover, secreted Fbn2 and Fbln2 known to interact in the formation of elastic fibers were found only in LH, thus indicating an inductive effect by LH, overcome by CIS. 

Exclusive of CIS + LH, Abracl, belonging to a novel family of low-molecular-weight proteins that increase actin dynamics, Akr1a1, a novel *S*-nitroso-glutathione reductase mediator of redox-based cellular signaling [40], Cdcc80, a member of the peroxiredoxin family of antioxidant enzymes [41], and Il4r, a component of the IL-4 receptor essential for tissue preservation and repair, were secreted [42]. As a matter of fact, the functions attributed to these compounds might contribute to protecting the cultured ovarian tissues and the oocytes within from CIS effects. 

Some proteins appearing in CTRL and CIS and never found in LH and CIS + LH, such as secreted Amh and Fam3c, could also have a role in the ongoing apoptotic processes occurring at a lower rate in CTRL and massively in CIS at this time. Amh is a member of the TGF-β family able to induce or prevent apoptosis according to the cell targets [43], while Fam3c, belonging to the FAM3 cytokine family, has been found to regulate the activity of various proteins, including Ras, STAT3, TGF-β, and LIFR [44]. Among the proteins common to CTRL and CIS, Agt was also present in the CIS medium at early culture times (see above).

Concerning secreted Adam10, Ctsl, Ifnar, Prdx5, Rbmx, and Rplp1, appearing at 24 h in CTRL and CIS but also in CIS + LH and never detected in LH, we can hypothesize an inhibitory action by the hormone lost in the presence of CIS and probably irrelevant for the deleterious effect of the drug on the ovarian tissue. Similar considerations can be drawn for secreted Adam12, S100a11, Cstb, and Hist2h4, also common to CTRL and CIS at this time but present at 12 h in CIS and CIS + LH (see above).

Hnrnpk, belonging to the subfamily of ubiquitously expressed heterogeneous nuclear ribonucleoproteins, and Rps21 and Rps6, ribosomal proteins with probable extra-ribosomal functions (see above Rps28), appeared only in CIS and CIS + LH, suggesting induction by CIS not influenced by the presence of LH and likely irrelevant for the proapoptotic effect of CIS on the ovarian tissues. Tuba1c, also present only in CIS and CIS + LH, was already detected in this latter group at 12 h (see above). 

The appearance of the potentially secreted Gsn, Ldha, Prdx2, and Prss23 in CTRL, LH, and CIS + LH, but not in CIS alone, suggests an inhibitory effect of the drug overcome by LH. Gsn is a Ca^2+^-dependent protein that modulates actin assembly and disassembly, exerting either proapoptotic or anti-apoptotic activity [45]; Ldha, apart from the undisputed role of the enzyme in cell metabolism and adaptation to unfavorable environmental or cellular conditions, participates in the regulation of cell death [46]; Prdx2 acts as an antioxidant and antiapoptotic enzyme in most cell types, including granulosa cells [47]; Prss23, a serine protease homologous to Prss35 (see above), was originally identified upregulated in mouse ovaries by estrogens and gonadotropins and possesses a probable role in preventing follicle atresia [48]. Interestingly, these activities are compatible with the antiapoptotic effect exerted by LH in the present system.

Secreted Ctgf, a matricellular protein of the CCN family of extracellular matrix-associated heparin-binding proteins, Dkk3, an antagonist of the canonical WNT signaling but with a role also in cytoskeleton organization, and Serping1, a highly glycosylated Serpin family G member involved in the regulation of the complement cascade, were shared by CTRL, LH, and CIS but absent in CIS + LH, implying a combined inhibitory effect of the compounds somehow beneficial for the cultured ovarian tissue. As a note, Dkk3 has been reported to play an important role in ovary development and granulosa cell tumorigenesis [34].

Finally, the potentially secreted Cfl1, a widely distributed intracellular actin-modulating protein, appeared in CIS, LH, and CIS + LH but not in CTRL, implying late induction by both compounds unrelated to the ovarian tissue status. Similarly, Ltbp4 is a protein that sequesters TGF-β and regulates its availability for binding to its receptor, which appeared in CIS and LH but not in CTRL and CIS + LH. 

The possible meaning of the trends of Isoc1 (appearing in CTRL and LH at this time and transiently present in CIS + LH at 2 h, never found in CIS alone), Htra1 (appearing in CTRL but already present in LH at 12 h and accumulated in CIS + LH throughout the culture, never found in CIS alone), Atp5a1 (coming out in LH and CIS + LH but present in CTRL at 12 h, never found in CIS alone) and Fbn1 (present in CTRL, LH, and CIS, already at 12 h, in CIS, but never found in CIS + LH) was discussed above.

## 4. Discussion

In the present work, our main aim was to characterize the extracellular in vitro microenvironment in which the oocytes within the primordial/primary follicles underwent apoptosis induced by CIS or were, for the most part, protected by concomitant administration of LH [2,3,6]. Moreover, we looked to confirm the ability of early postnatal ovaries to respond to LH stimulation.

Concerning this last point, the differences in protein expression in the culture media of the LH-treated group compared to the CTRL emphasize the responsiveness of the ovarian tissue at early developmental stages [2,6]. Among those of particular interest, Ltbp1 belonging to the family of TGF-β binding proteins, the collagenic Colla2f and Col5a1, Grn, an endoplasmic reticulum stress-responsive factor, were present only in LH medium at 12 h, and Fbn2 and Fbln2, known to interact in the formation of elastic fibers, were found only in LH at 24 h. Conversely, several proteins playing a variety of roles, such as Adam10, Adam12, Cstb, Ctsl, Ifnar2, Pkm, Prdx5, Rbmx, Rplp1, Fam3c, and Zp1, were never found in the LH medium. The meaning of such inductive or repressive action of LH at these early ovary developmental stages deserves further investigation.

About the first aim, the present data provide new clues about how CIS and LH might modify the extracellular microenvironment of the ovarian tissues favoring apoptosis or counteracting the CIS proapoptotic effects, respectively. Our previous investigation did not reveal morphological differences or significant expression of apoptotic and DNA damage markers in ovarian tissues treated with CIS in comparison to the other groups at 2 h; however, these markers, paralleled with morphological signs of degeneration, became apparent in cultured ovarian tissue exposed to CIS from 12 h onwards [2]. 

In this regard, collectively, the present results indicated that the apoptotic conditions generated by CIS in the cultured ovarian tissues and/or oocytes is reflected in distinct changes of the extracellular microenvironment. These changes became evident mainly from 12 h onwards and were characterized by inhibited or decreased release of a variety of compounds, such as Htra1 and Prss23, serine proteases highly expressed in the ovary and modulated by estrogens, and LH [13,48], and factors that usually sustain cell survival, such as the antioxidant and antiapoptotic Prdx2 and Hbat1, the metabolic regulators Ldha and Pkm, and the regulator of apoptotic pathways Tmsb4x [20,21,22,23,46,47]. At the same time, CIS appeared to increase the release of several proteins that generally favor apoptosis, such as Amh and the ECM protein Fbn1 reported to be able to induce this process in the follicular cells [43,49,50], the cytokine Fam3c [44], Ecm1 [36], the 14-3-3 family hub protein Ywhaz [39], and the protease Ctsb [51]. The drug, however, seemed also able to induce the release of proteins usually exerting antiapoptotic effects, such as Adam12 [52], Agt [53,54,55], Tpt1 [37], Cstb [28], and Tuba1c [29], probably representing the attempt of the tissue to counteract the death processes, and of Ahnak, an unusual desmoyokin scaffolding protein with a role in diverse processes, such as cell structure and migration, calcium channel regulation, and tumor metastasis [35]. As a note, Tuba1c has been recently proposed as ovarian cancer marker [56].

**Figure 7:**
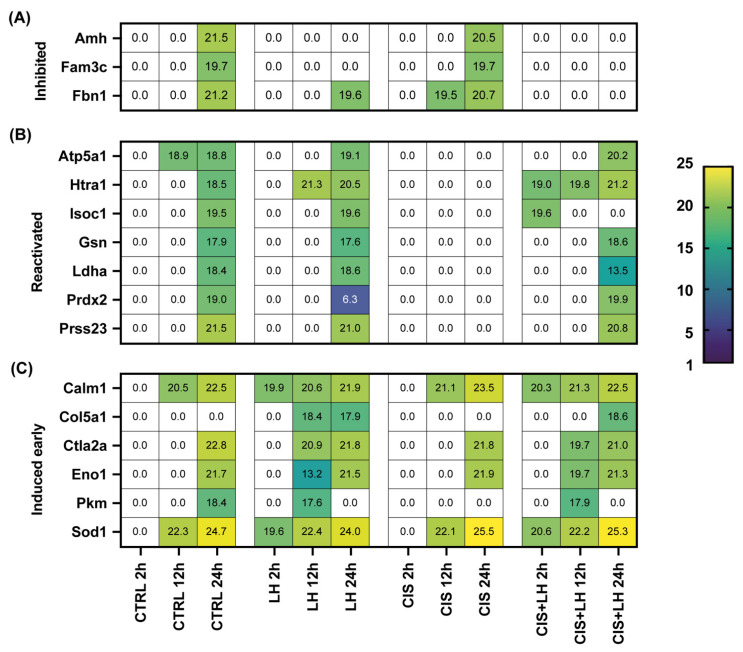
Heatmap comparison of the Mean log_2_ iBAQ values of metabolites hypothetically implicated in the protective effect of LH against CIS-induced apoptosis. LH was able to (**A**) inhibit the expression and/or the release in the microenvironment of probable proapoptotic factors induced by CIS in the ovarian tissues (Inhibited); (**B**) reactivate those of several antiapoptotic factors inhibited by CIS (Reactivated); (**C**) anticipate the induction of other (Induced early).

The actions of CIS and LH were clearly reciprocally influenced when the compounds were used together offering some clues as to how the hormone influences the composition of the extracellular environment in order to protect primarily the oocytes enclosed in primordial/primary follicles from apoptosis [2,3,6]. As a matter of fact, it is likely that such LH protective effect occurs through multiple ways. In our previous work [2], we postulated that LH stimulates somatic cells to transfer small molecules (for example, cAMP and cGMP) to the oocyte via gap junctions and to release compounds in the extracellular milieu (for example, Kit ligand (KL)), making oocytes able to activate antiapoptotic pathways and repair DNA damage induced by the drug. Although compounds released in the extracellular milieu at relatively low concentrations which might be able to exert these effects and may have been missed by our analysis (KL itself was not detected in our assay), the present results provide useful indications about a number of pro- and anti-apoptotic molecules or pathways that LH is able to stimulate in the ovarian tissues, likely of the somatic compartment, upon CIS exposure. Among these molecules, particularly interesting were the inhibition of expression and/or release of proapoptotic factors induced by CIS, such as Amh, Fam3c, and Fbn1, and the reactivation of expression and/or release of antiapoptotic factors inhibited by CIS, such as Atp5a1, Htra1, Isoc1, Gsn, Ldha, Prdx2, and Prss23 (see above), or de novo stimulation of others, such as Calm1, Col5a1, Ctla2a, Eno1, Pkm, and, interestingly, Sod1, an isoform of a superoxide dismutase one of the major antioxidant enzymes (Figure 7, Table 1 and Table 2). Future studies will determine if and how these proteins are able to preserve the oocytes from the proapoptotic action of CIS. Moreover, some of the proteins identified and analyzed in this work (Actg1, Atp5a1, Calm1, Cstb, Dbi, Eef1a1, Gapdh, Hist1h1b, Hist1h1d, Hist1h1e, Hist2h4, Hnrnpk, Hspe1, Isoc1, Nme1, Pabc1, Pgam1, Pkm, Ppic, Rpl6, Rplp1, Rplp2, Rps21, Rps28, Tagln2, Tmsb10, Tmsb4x, Tpi1, Tpt1, Tuba1c, Ube2n, Uchl1, Vim, Ybx1, Ywhaz) are intracellular proteins, potentially released into the culture medium through exosome. Additional studies, however, are needed to verify their origin.

Besides unraveling the action mechanisms of CIS and LH on the developing ovaries, the present data might represent a valuable resource for future studies aimed at clarifying the effects and identifying biomarkers of the action of these compounds on these essential reproductive organs.

The possibility that the fertoprotective effect of LH on ovaries exposed to certain chemotherapies may influence the levels of estrogens or progesterone in patients should be investigated in future studies. Such a possible consequence is relevant since estrogens and progesterone seem to exert opposite effects on the development and progression of some types of tumors. In particular, besides the well-established notion that sustained exposure to exogenous estrogen is a risk factor for various cancers, recent studies indicate that progestin exposure significantly reduces the incidence of ovarian, pancreatic, and lung cancers in addition to endometrial cancer in women (for a recent review, see Li et al., 2020 [57]). This latter action suggests that a forthcoming careful use of LH as a fertoprotective could be associated with a protective action against cancer development and progression.

## Figures and Tables

**Table 1:** Expression in CTRL and LH groups of metabolites hypothetically implicated in the protective effect of LH against CIS-induced apoptosis. Mean log_2_ iBAQ values and SD are reported. LH was able to inhibit the expression and/or the release in the microenvironment of probable proapoptotic factors induced by CIS in the ovarian tissues (Inhibited); reactivate those of several antiapoptotic factors inhibited by CIS (Reactivated); anticipate the induction of other (Induced early).

		CTRL			LH		
		2 h	12 h	24 h	2 h	12 h	24 h
Inhibited	Amh	0 ± 0	0 ± 0	21.41 ± 0.76	0 ± 0	0 ± 0	0 ± 0
	Fam3c	0 ± 0	0 ± 0	19.69 ± 0.48	0 ± 0	0 ± 0	0 ± 0
	Fbn1	0 ± 0	0 ± 0	21.18 ± 0.69	0 ± 0	0 ± 0	19.65 ± 0.88
Reactivated	Atp5a1	0 ± 0	18.94 ± 1.59	18.84 ± 1.55	0 ± 0	0 ± 0	19.01 ± 1.96
	Htra1	0 ± 0	0 ± 0	18.48 ± 0.86	0 ± 0	21.03 ± 0.31	20.47 ± 1.98
	Isoc1	0 ± 0	0 ± 0	19.05 ± 0.25	0 ± 0	0 ± 0	19.62 ± 0.96
	Gsn	0 ± 0	0 ± 0	17.94 ± 0.98	0 ± 0	0 ± 0	17.58 ± 0.51
	Ldha	0 ± 0	0 ± 0	18.37 ± 0.02	0 ± 0	0 ± 0	18.63 ± 0.68
	Prdx2	0 ± 0	0 ± 0	18.99 ± 0.81	0 ± 0	0 ± 0	6.25 ± 0.0
	Prss23	0 ± 0	0 ± 0	21.53 ± 0.18	0 ± 0	0 ± 0	20.98 ± 0.88
Induced early	Calm1	0 ± 0	20.53 ± 0.33	22.48 ± 1.16	19.89 ± 0.20	20.56 ± 0.57	21.81 ± 0.80
	Col5a1	0 ± 0	0 ± 0	0 ± 0	0 ± 0	18.39 ± 0.25	17.90 ± 1.08
	Ctla2a	0 ± 0	0 ± 0	22.84 ± 0.47	0 ± 0	20.87 ± 0.25	21.84 ± 0.63
	Eno1	0 ± 0	0 ± 0	21.69 ± 0.58	0 ± 0	13.24 ± 0.13	21.52 ± 0.19
	Pkm	0 ± 0	0 ± 0	18.39 ± 0.75	0 ± 0	17.63 ± 0.61	0 ± 0
	Sod1	0 ± 0	22.03 ± 1.53	24.68 ± 0.96	19.63 ± 0.32	22.40 ± 1.07	23.99 ± 1.44

**Table 2:** Expression in CIS and CIS + LH of metabolites hypothetically implicated in the protective effect of LH against CIS-induced apoptosis. Mean log_2_ iBAQ values and SD are reported. LH was able to inhibit the expression and/or the release in the microenvironment of probable proapoptotic factors induced by CIS in the ovarian tissues (Inhibited); reactivate those of several antiapoptotic factors inhibited by CIS (Reactivated); anticipate the induction of other (Induced early).

		CIS			CIS + LH		
		2 h	12 h	24 h	2 h	12 h	24 h
Inhibited	Amh	0 ± 0	0 ± 0	20.52 ± 0.77	0 ± 0	0 ± 0	0 ± 0
	Fam3c	0 ± 0	0 ± 0	19.69 ± 0.68	0 ± 0	0 ± 0	0 ± 0
	Fbn1	0 ± 0	19.50 ± 1.39	20.70 ± 1.06	0 ± 0	0 ± 0	0 ± 0
Reactivated	Atp5a1	0 ± 0	0 ± 0	0 ± 0	0 ± 0	0 ± 0	20.11 ± 0.48
	Htra1	0 ± 0	0 ± 0	0 ± 0	18.98 ± 1.62	19.71 ± 2.97	21.15 ± 0.60
	Isoc1	0 ± 0	0 ± 0	0 ± 0	19.63 ± 0.06	0 ± 0	0 ± 0
	Gsn	0 ± 0	0 ± 0	0 ± 0	0 ± 0	0 ± 0	18.59 ± 1.93
	Ldha	0 ± 0	0 ± 0	0 ± 0	0 ± 0	0 ± 0	13.45 ± 0.03
	Prdx2	0 ± 0	0 ± 0	0 ± 0	0 ± 0	0 ± 0	19.81 ± 0.76
	Prss23	0 ± 0	0 ± 0	0 ± 0	0 ± 0	0 ± 0	20.80 ± 0.30
Induced early	Calm1	0 ± 0	21.11 ± 1.33	23.46 ± 0.30	20.28 ± 0.47	21.30 ± 1.32	22.44 ± 0.77
	Col5a1	0 ± 0	0 ± 0	0 ± 0	0 ± 0	0 ± 0	18.60 ± 0.48
	Ctla2a	0 ± 0	0 ± 0	21.78 ± 0.72	0 ± 0	19.75 ± 2.01	20.98 ± 1.62
	Eno1	0 ± 0	0 ± 0	21.86 ± 0.43	0 ± 0	19.70 ± 1.37	21.26 ± 1.02
	Pkm	0 ± 0	0 ± 0	18.39 ± 0.75	0 ± 0	17.63 ± 0.61	0 ± 0
	Sod1	0 ± 0	22.03 ± 1.53	24.68 ± 0.96	19.63 ± 0.32	22.40 ± 1.07	23.99 ± 1.44

## Data Availability

Data are contained within the article or Supplementary Materials.

## Supplementary Materials

The following supporting information can be downloaded at: https://www.mdpi.com/article/10.3390/cells11071208/s1, Figure S1: LH protective effect against CIS-induced apoptosis of POs was confirmed; Figure S2: Venn diagram analyses of FCM composition after treatment with/out CIS and LH for 2, 12, and 24 h; Figure S3: Venn diagram analyses of upregulated proteins in the shared groups after treatment with/out CIS and LH for 2, 12, and 24 h. Data S1: Proteome dataset; Table S1: List of proteins identified by iBAQ along with their localization; Table S2: Metabolites amount trend during culture time; Table S3: Shared proteins between indicated groups.
