# Supplementary material for: Analysis of Secreted Proteins from Prepubertal Ovarian Tissues Exposed In Vitro to Cisplatin and LH
Source: Cells. 2022 Apr 3;11(7):1208. doi: 10.3390/cells11071208 (PMC8997822; doi:10.3390/cells11071208)
Supplement: Supplementary file 1 [file cells-11-01208-s001.zip › cells-1626341-supplementary.pdf]

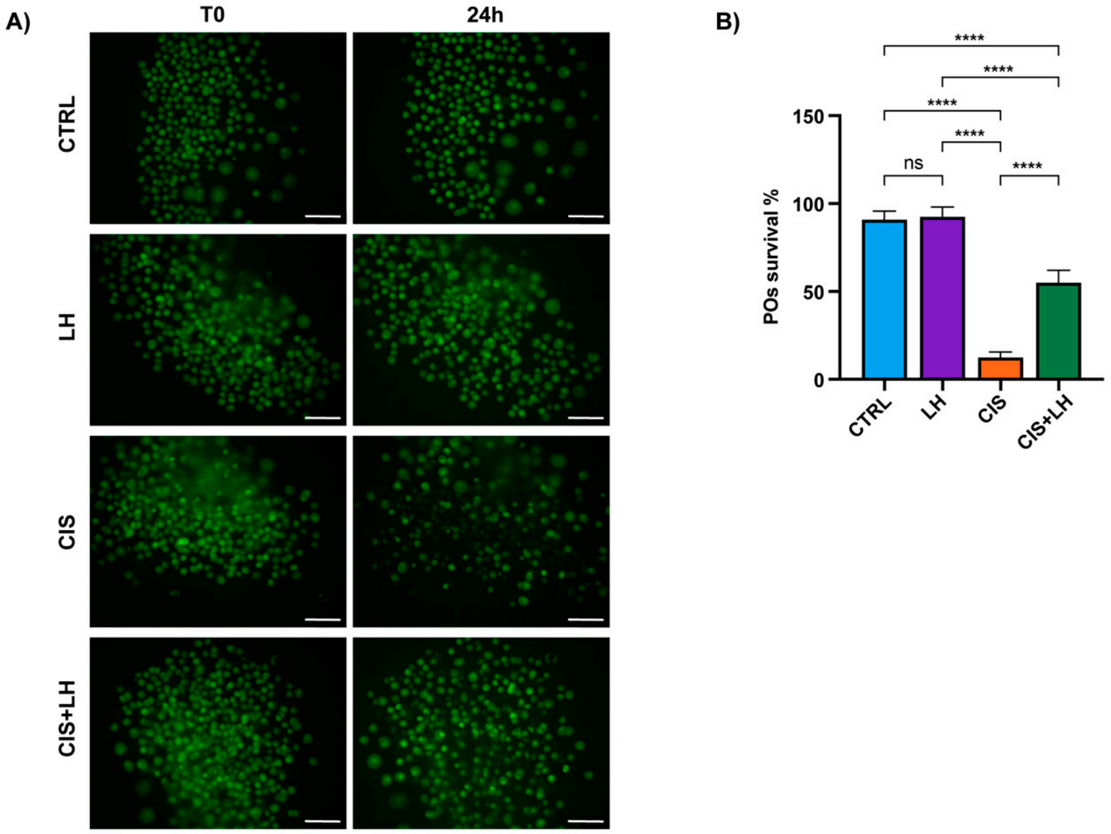

Figure S1. LH protective effect against CIS-induced apoptosis of POs was confirmed. a) Microphotographs of ovarian fragments from P4 ovaries of p-18 c-Kit/GFP mice cultured for 4 days (T0) and incubated for 24 hrs in the presence of the indicated compounds. Scale bar = 100µm. b) Percentage of healthy POs after 24 hrs of culture; only GFP-positive oocytes with a diameter < 20µm were considered for the analysis. The data confirmed the drastic reduction of the healthy POs number after CIS treatment compared to CTRL and the ability of LH pre-treatment to support survival. Data are represented as mean ± SD. Statistical differences between indicated groups are reported ****$p<0.0001$.

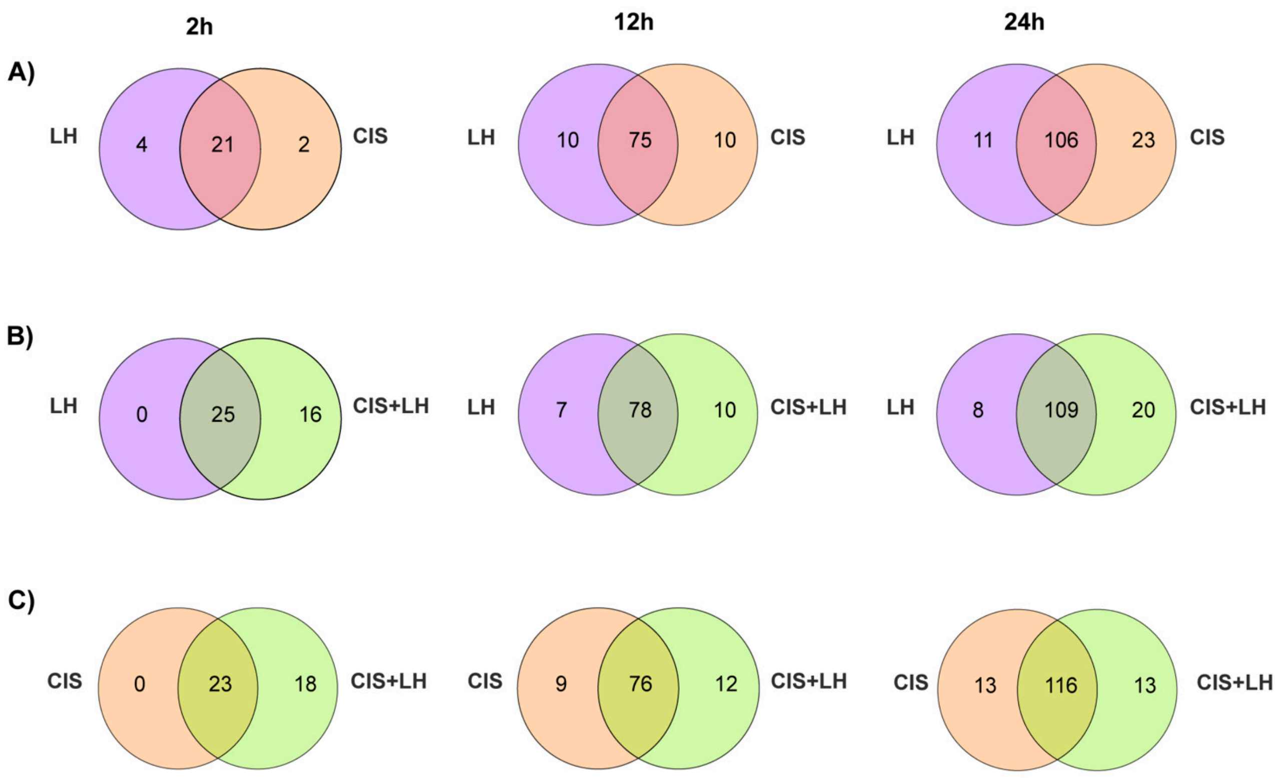

Figure S2. Venn diagram analyses of FCM composition after treatment with/out CIS and LH for 2, 12 or 24 hrs. a) Overlaps between LH and CIS at 2, 12 and 24 hrs of treatment. b) Overlaps between LH and CIS+LH at 2, 12 and 24 hrs of treatment. c) Overlaps between CIS and CIS+LH at 2, 12 and 24 hrs of treatment.

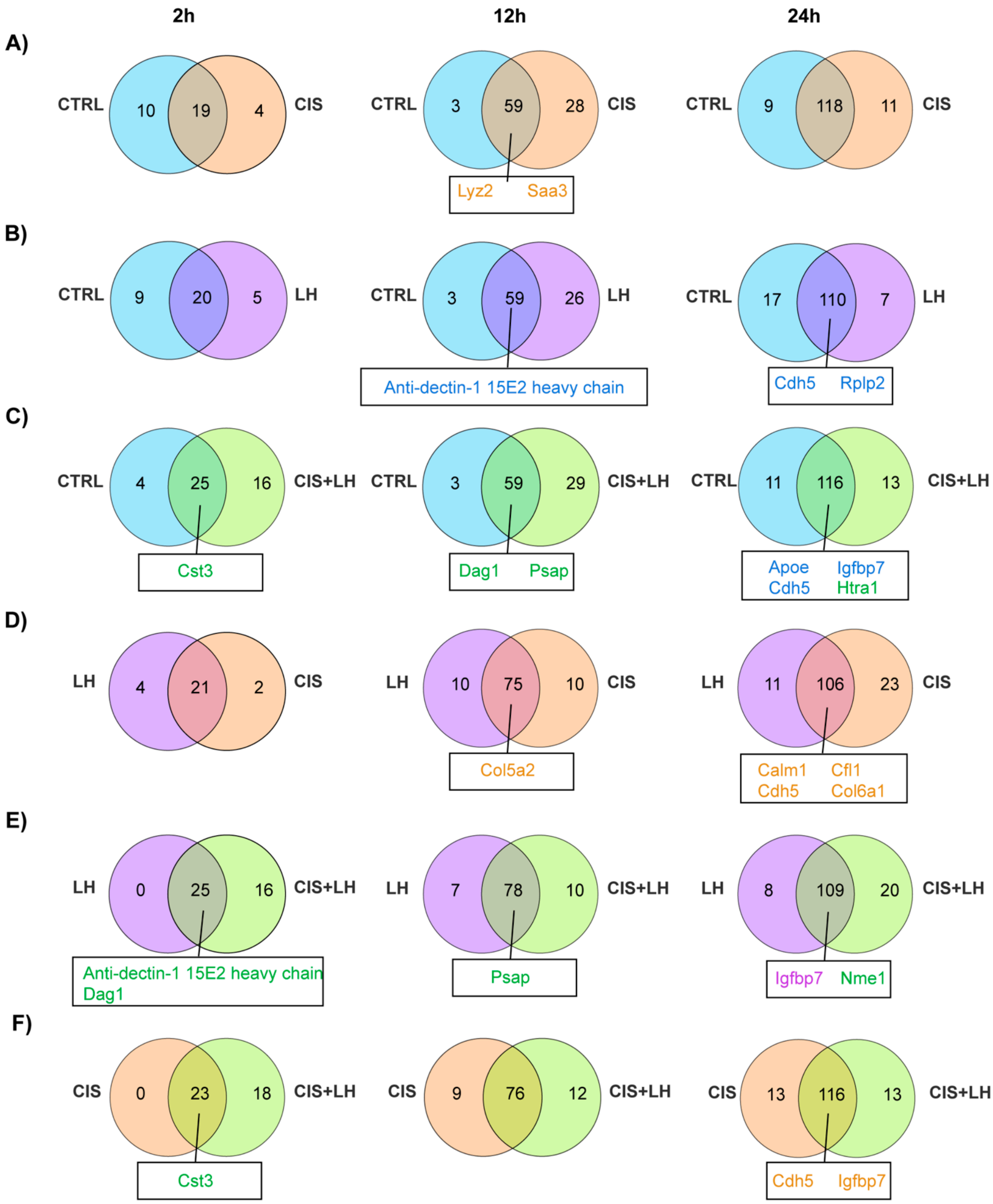

Figure S3. Venn diagram analyses of FCM composition after treatment with/out CIS and LH for 2, 12 or 24 hrs. Upregulated proteins in the shared groups are reported. Upregulated in CTRL group in blue; upregulated in LH group in purple; upregulated in CIS group in orange; upregulated in CIS+LH group in green. a) Overlaps between CTRL and CIS and CIS+LH at 2, 12 and 24 hrs of treatment. b) Overlaps between CTRL and LH at 2, 12 and 24 hrs of treatment. c) Overlaps between CTRL and CIS+LH at 2, 12 and 24 hrs of treatment. d) Overlaps between LH and CIS and CIS+LH at 2, 12 and 24 hrs of treatment. e) Overlaps between LH and CIS+LH at 2, 12 and 24 hrs of treatment. f) Overlaps between CIS and CIS+LH at 2, 12 and 24 hrs of treatment.

Table S1. List of proteins identified by iBAQ along with their localization. The majority of proteins (114/170, shown in blue) were identified as secretory proteins whereas 56/170 proteins are predicted as intracellular. However, 35 of these could be secreted via extravescicles (EVs, shown in green), so only 21/170 (shown in orange) can be considered exclusively intracellular.

PROTEIN NAMES	GENE NAMES	LOCALIZATION
**Costars family protein ABRACL**	Abracl	Secreted
**Actin, cytoplasmic 2**	Actg1	EVs/Intracellular
**Disintegrin and metalloproteinase domain-containing protein 10**	Adam10	Secreted/EVs/Intracellular
**Disintegrin and metalloproteinase domain-containing protein 12**	Adam12	Secreted/Intracellular
**A disintegrin and metalloproteinase with thrombospondin motifs 1**	Adamts1	Secreted/Intracellular
**A disintegrin and metalloproteinase with thrombospondin motifs 2**	Adamts2	Secreted
**Angiotensinogen;**	Agt	Secreted
**Desmoyokin**	Ahnak	Secreted/Intracellular
**Alcohol dehydrogenase [NADP(+)]**	Akr1a1	Secreted/EVs/Intracellular
**Serum albumin**	Alb	Secreted/EVs
**Aly/REF export factor 2**	Alyref2	Intracellular
**Muellerian-inhibiting factor**	Amh	Secreted
**Anti-dectin-1 15E2 heavy chain**	Anti-dectin-1 15E2 heavy chain	Secreted
**Anti-dectin-1 15E2 light chain**	Anti-dectin-1 15E2 light chain	Secreted
**Apolipoprotein A-I**	Apoa1	Secreted
**Apolipoprotein E**	Apoe	Secreted/EVs
**Copper transport protein ATOX1**	Atox1	Secreted/Intracellular
**ATP synthase subunit alpha**	Atp5a1	EVs/Intracellular
**V-type proton ATPase subunit S1**	Atp6ap1	Intracellular
**Beta-2-microglobulin**	B2m	Secreted/EVs/Intracellular
**Flavin reductase (NADPH)**	Blvrb	Intracellular

**Bone morphogenetic protein 1**	Bmp1	Secreted/EVs
**Calmodulin**	Calm1	EVs/Intracellular
**Coiled-coil domain-containing protein 80**	Ccdc80	Secreted
**Cadherin-11**	Cdh11	Intracellular
**Cadherin-13**	Cdh13	Secreted/Intracellular
**Cadherin-2**	Cdh2	Intracellular
**Cadherin-5**	Cdh5	Secreted
**Cofilin-1**	Cfl1	Secreted/EVs/Intracellular
**Clusterin**	Clu	Secreted/EVs/Intracellular
**Collagen alpha-1(XV) chain**	Col15a1	Secreted
**Collagen alpha-1(XVIII) chain**	Col18a1	Secreted
**Collagen alpha-1(I) chain**	Col1a1	Secreted/EVs
**Collagen alpha-2(I) chain**	Col1a2	Secreted/EVs
**Collagen alpha-2(I) chain (fragment)**	Col1a2f	Secreted
**Collagen alpha-1(III) chain**	Col3a1	Secreted
**Collagen alpha-1(IV) chain**	Col4a1	Secreted
**Collagen alpha-2(IV) chain**	Col4a2	Secreted
**Collagen alpha-1(V) chain**	Col5a1	Secreted/EVs
**Collagen alpha-2(V) chain**	Col5a2	Secreted
**Collagen alpha-1(VI) chain**	Col6a1	Secreted/EVs
**Collagen alpha-3(VI) chain**	Col6a3	Secreted
**Collectin-12**	Colec12	Secreted
**Cytochrome c oxidase subunit 5A**	Cox5a	Intracellular
**Carboxypeptidase E**	Cpe	Secreted/EVs

**Cystatin-C**	Cst3	Secreted
**Cystatin-B**	Cstb	EVs/Intracellular
**Connective tissue growth factor**	Ctgf	Secreted
**Protein CTLA-2-alpha**	Ctla2a	Secreted
**Cathepsin B**	Ctsb	Secreted/EVs/Intracellular
**Cathepsin L1**	Ctsl	Secreted/EVs
**Dystroglycan**	Dag1	Secreted/EVs
**Acyl-CoA-binding protein**	Dbi	EVs/Intracellular
**D-dopachrome decarboxylase**	Ddt	Secreted/Intracellular
**Dickkopf-related protein 3**	Dkk3	Secreted
**Extracellular matrix protein 1**	Ecm1	Secreted
**Elongation factor 1-alpha**	Eef1a1	EVs/Intracellular
**Alpha-enolase**	Eno1	Secreted/EVs/Intracellular
**Elastin**	Eln	Secreted
**Protein FAM3C**	Fam3c	Secreted
**Fibulin-2**	Fbln2	Secreted/EVs
**Fibrillin-1**	Fbn1	Secreted
**Fibrillin-2**	Fbn2	Secreted
**Peptidyl-prolyl cis-trans isomerase**	Fkbp1a	Intracellular
**Fibronectin**	Fn1	Secreted/EVs
**Follistatin-related protein 1**	Fstl1	Secreted
**Glyceraldehyde-3-phosphate dehydrogenase**	Gapdh	EVs/Intracellular
**Ganglioside GM2 activator**	Gm2a	Intracellular
**Granulins**	Grn	Secreted

**Gelsolin**	Gsn	Secreted/EVs/Intracellular
**Glutathione S-transferase A4**	Gsta4	Intracellular
**Hemoglobin subunit alpha**	Hba	Secreted/Intracellular
**Alpha-globin**	Hbat1	Secreted/Intracellular
**Hemoglobin subunit beta-2**	Hbb-b2	Secreted/EVs/Intracellular
**Protein HEG homolog 1**	Heg1	Intracellular
**Protein HEG homolog 1**	Heg1	Intracellular
**Histone H1.5**	Hist1h1b	EVs/Intracellular
**Histone H1.3**	Hist1h1d	EVs/Intracellular
**Histone H1.4**	Hist1h1e	EVs/Intracellular
**Histone H2A**	Hist1h2al	Intracellular
**Histone H4**	Hist2h4	EVs/Intracellular
**Heterogeneous nuclear ribonucleoprotein K**	Hnrnpk	EVs/Intracellular
**Hemopexin**	Hpx	Secreted
**10 kDa heat shock protein**	Hspe1	EVs/Intracellular
**Basement membrane-specific heparan sulfate proteoglycan core protein**	Hspg2	Secreted/EVs
**Serine protease HTRA1**	Htra1	Secreted/EVs
**Interferon alpha/beta receptor 2**	Ifnar2	Secreted
**Insulin-like growth factor I**	Igf1	Secreted
**Insulin-like growth factor II**	Igf2	Secreted
**Insulin-like growth factor-binding protein 2**	Igfbp2	Secreted
**Insulin-like growth factor-binding protein 3**	Igfbp3	Secreted
**Insulin-like growth factor-binding protein 4**	Igfbp4	Secreted
**Insulin-like growth factor-binding protein 5**	Igfbp5	Secreted

**Insulin-like growth factor-binding protein 7**	Igfbp7	Secreted
**Soluble interleukin-4 receptor subunit alpha**	Il4r	Secreted/Intracellular
**Inhibin alpha chain**	Inha	Secreted
**Isochorismatase domain-containing protein 1**	Isoc1	EVs/Intracellular
**Inter-alpha-trypsin inhibitor heavy chain H5**	Itih5	Secreted
**Integral membrane protein 2B**	Itm2b	Secreted
**Keratin 76**	Krt76	Intracellular
**L-lactate dehydrogenase A chain**	Ldha	Secreted/EVs/Intracellular
**L-lactate dehydrogenase B chain**	Ldhb	Secreted/Intracellular
**Galectin-1**	Lgals1	Secreted/EVs/Intracellular
**Protein-lysine 6-oxidase**	Lox	Secreted/EVs/Intracellular
**Lysyl oxidase homolog 1**	Loxl1	Secreted
**Latent-transforming growth factor beta-binding protein 1**	Ltbp1	Secreted/EVs
**Latent-transforming growth factor beta-binding protein 4**	Ltbp4	Secreted/EVs
**Lumican**	Lum	Secreted
**Lysozyme**	Lyz2	Secreted
**Macrophage migration inhibitory factor**	Mif	Secreted/EVs
**Matrix metalloproteinase-14**	Mmp14	Secreted/Intracellular
**72 kDa type IV collagenase**	Mmp2	Secreted
**NEDD8**	Nedd8	Secreted/Intracellular
**Nidogen-2**	Nid2	Secreted/Intracellular
**Nucleoside diphosphate kinase**	Nme1	EVs/Intracellular
**Nucleobindin-1**	Nucb1	Secreted/EVs/Intracellular
**Nuclear transport factor 2**	Nutf2	Intracellular

**Polyadenylate-binding protein**	Pabpc1	EVs/Intracellular
**Platelet-activating factor acetylhydrolase IB subunit beta**	Pafah1b2	Intracellular
**Protein deglycase DJ-1**	Park7	Intracellular
**Phosphatidylethanolamine-binding protein 1**	Pebp1	Secreted/EVs/Intracellular
**Phosphoglycerate mutase 1**	Pgam1	EVs/Intracellular
**Pyruvate kinase PKM**	Pkm	EVs/Intracellular
**Peptidyl-prolyl cis-trans isomerase**	Ppia	Secreted/EVs/Intracellular
**Peptidyl-prolyl cis-trans isomerase**	Ppic	EVs/Intracellular
**Peroxiredoxin-1**	Prdx1	Secreted/EVs/Intracellular
**Peroxiredoxin-2**	Prdx2	Secreted/EVs/Intracellular
**Peroxiredoxin-5**	Prdx5	Secreted/Intracellular
**Peroxiredoxin-6**	Prdx6	Secreted/Intracellular
**Major prion protein**	Prnpb	Secreted/Intracellular
**Serine protease 23**	Prss23	Secreted/EVs
**Inactive serine protease 35**	Prss35	Secreted
**Prosaposin**	Psap	Secreted/EVs
**Polypyrimidine tract-binding protein 1**	Ptbp1	Intracellular
**Peroxidasin homolog**	Pxdn	Secreted
**Sulfhydryl oxidase 1**	Qsox1	Secreted
**RNA binding motif protein, X-linked-like-1**	Rbmx	Secreted/Intracellular
**Retinol-binding protein 1**	Rbp1	Intracellular
**Ribonuclease 4**	Rnase4	Secreted
**60S ribosomal protein L6**	Rpl6	EVs/Intracellular
**60S acidic ribosomal protein P1**	Rplp1	EVs/Intracellular

**60S acidic ribosomal protein P2**	Rplp2	EVs/Intracellular
**40S ribosomal protein S21**	Rps21	EVs/Intracellular
**40S ribosomal protein S28**	Rps28	EVs/Intracellular
**Protein S100-A11**	S100a11	Secreted/Intracellular
**Serum amyloid A-3 protein**	Saa3	Secreted
**Antithrombin-III**	Serpinc1	Secreted/EVs
**Pigment epithelium-derived factor**	Serpinf1	Secreted/EVs
**Plasma protease C1 inhibitor**	Serping1	Secreted
**Protein shisa-5**	Shisa5	Intracellular
**Superoxide dismutase [Cu-Zn]**	Sod1	Secreted/EVs/Intracellular
**SPARC**	Sparc	Secreted
**Transgelin**	Tagln	Intracellular
**Transgelin-2**	Tagln2	EVs/Intracellular
**Serotransferrin**	Tf	Secreted
**Thymosin beta-10**	Tmsb10	EVs/Intracellular
**Thymosin beta-4**	Tmsb4x	EVs/Intracellular
**Triosephosphate isomerase**	Tpi1	EVs/Intracellular
**Translationally-controlled tumor protein**	Tpt1	EVs/Intracellular
**Tubulin alpha-1C chain**	Tuba1c	EVs/Intracellular
**Ubiquitin-60S ribosomal protein L40**	Ubc	Intracellular
**Ubiquitin-conjugating enzyme E2 N**	Ube2n	EVs/Intracellular
**Ubiquitin carboxyl-terminal hydrolase isozyme L1**	Uchl1	EVs/Intracellular
**Vimentin**	Vim	EVs/Intracellular
**Y-box-binding protein 1**	Ybx1	EVs/Intracellular

**14-3-3 protein zeta/delta**	Ywhaz	EVs/Intracellular
**Coiled-coil-helix-coiled-coil-helix domain-containing protein 2**	Zbed5	Intracellular
**Zona pellucida sperm-binding protein 1**	Zp1	Secreted
**Zona pellucida sperm-binding protein 2**	Zp2	Secreted
**Zona pellucida sperm-binding protein 3**	Zp3	Secreted

Table S2. Metabolites amount trend during culture time. Appeared: proteins that are not expressed at 2 hrs but appear during culture time (12 hrs in red, 24 hrs in black). Disappeared: proteins whose expression disappears during the culture period. Accumulated: proteins already expressed at 2 hrs time point and whose expression increases during the culture time. Decreased: proteins expressed at 2 hrs of culture and whose expression decreases during the 24 hrs of analysis Variable: proteins with variable expression mode over time. Stable: proteins whose expression does not change during the period of culture analysed.

**CTRL**	**Appeared**	Adam10, Adam12, Adamts1, Adamts2, Agt, Amh, Apoe, Atox1, Atp5a1, Bmp1, Calm1, Cdh13, Cdh5, Clu, Col15a1, Col18a1, Col1a2f, Col4a1, Col4a2, Col5a2, Col6a1, Col6a3, Colec12, Cpe, Cstb, Ctgf, Ctla2a, Ctsb, Ctsl, Dbi, Ddt, Dkk3, Eef1a1, Eno1, Eln, Fam3c, Fbn1, Fn1, Fstl1, Grn, Gsn, Hbb-b2, Hist1h1b, Hist2h4, Hspe1, Hspg2, Htra1, Ifnar2, Igf2, Igfbp2, Igfbp3, Igfbp4, Igfbp5, Igfbp7, Inha, Isoc1, Itih5, Ldha, Ldhb, Lox, Loxl1, Ltbp1, Lum, Mmp14, Mmp2, Nedd8, Nid2, Nme1, Nucb1, Pabpc1, Pebp1, Pgam1, Pkm, Ppic, Prdx1, Prdx2, Prdx5, Prdx6, Prnpb, Prss23, Prss35, Psap, Pxdn, Qsox1, Rbmx, Rnase4, Rplp1, Rplp2, Rps28, S100a11, Serping1, Sod1, Tagln2, Tmsb10, Tpi1, Ube2n, Uchl1, Vim, Zp1, Zp2, Zp3	101
	**Disappeared**	Alb, Apoa1, Hba, Serpinc1	4
	**Accumulated**	B2m, Col1a1, Col1a2, Col3a1, Cst3, Dag1, Gapdh, Itm2b, Lgals1, Lyz2, Mif, Ppia, Saa3, Sparc, Tmsb4x,	15
	**Decreased**	Anti-dectin-1 15E2 heavy chain, Hpx	2
	**Stable**	Anti-dectin-1 15E2 light chain, Hist1h1d, Hist1h1e	3
	**Variable**	Actg1, Cfl1, Hbat1, Igf1, Serpinf1, Tf, Ybx1	7
**LH**	**Appeared**	Adamts1, Adamts2, Apoa1, Apoe, Atox1, Atp5a1, Bmp1, Cdh5, Cfl1, Clu, Col15a1, Col18a1, Col1a2f, Col3a1, Col4a1, Col4a2, Col5a1, Col5a2, Col6a1, Col6a3, Colec12, Cpe, Ctgf, Ctla2a, Ctsb, Dbi, Ddt, Dkk3, Eef1a1, Eln, Eno1, Fbln2, Fbn1, Fbn2, Fn1, Fstl1, Grn, Gsn, Hist1h1e, Hspe1, Hspg2, Htra1, Igf1, Igfbp2, Igfbp3, Igfbp4, Igfbp5, Igfbp7, Inha, Isoc1, Itih5, Ldha, Ldhb, Lox, Loxl1, Ltbp1, Ltbp4, Lum, Lyz2, Mmp14, Mmp2, Nedd8, Nid2, Nme1, Nucb1, Pabpc1, Pebp1, Pgam1, Ppic, Prdx1, Prdx2, Prdx6, Prnpb, Prss23, Prss35, Psap, Pxdn, Qsox1, Rnase4, Rplp2, Rps28, Saa3, Serping1, Tagln2, Tmsb10, Tpi1, Ube2n, Uchl1, Vim, Ybx1, Zp2, Zp3	92
	**Disappeared**		0
	**Accumulated**	Actg1, B2m, Calm1, Col1a1, Col1a2, Cst3, Dag1, Igf2, Itm2b, Lgals1, Mif, Ppia, Serpinf1, Sod1, Sparc, Ubc	15
	**Decreased**		0
	**Stable**	Anti-dectin-1 15E2 heavy chain, Anti-dectin-1 15E2 light chain, Hist1h1b, Hist1h1d, Hpx, Tf	6
	**Variable**	Gapdh, Hbat1, Hbb-b2, Hist2h4, Pkm, S100a11, Tmsb4x	8
**CIS**	**Appeared**	Adam10, Adam12, Adamts1, Adamts2, Ahnak, Amh, Atox1, Bmp1, Calm1, Cdh5, Cfl1, Clu, Col15a1, Col18a1, Col1a2f, Col3a1, Col4a1, Col4a2, Col5a2, Col6a1, Col6a3, Colec12, Cpe, Cstb, Ctgf, Ctla2a, Ctsb, Ctsl, Dbi, Ddt, Dkk3, Ecm1, Eef1a1, Eln, Eno1, Fam3c, Fbn1, Fn1, Fstl1, Grn, Hbat1, Hnrnpk, Hspe1, Hspg2, Ifnar2, Igf1, Igfbp2, Igfbp3, Igfbp4, Igfbp5, Igfbp7, Inha, Itih5, Ldhb, Lox, Loxl1, Ltbp1, Ltbp4, Lum, Lyz2, Mmp14, Mmp2, Nedd8, Nid2, Nme1, Nucb1, Pabpc1, Pebp1, Pgam1, Ppic, Prdx1, Prdx5, Prdx6, Prnpb, Prss35, Psap, Pxdn, Qsox1, Rbmx, Rnase4, Rpl6, Rplp1, Rplp2, Rps21, Rps28, S100a11, Serping1, Sod1, Tagln2, Tmsb4x, Tpi1, Tpt1, Tuba1c, Ube2n, Uchl1, Vim, Ywhaz, Zp1, Zp2, Zp3	100
	**Disappeared**		0
	**Accumulated**	Actg1, B2m, Col1a1, Col1a2, Cst3, Dag1, Gapdh, Igf2, Itm2b, Lgals1, Mif, Ppia, Serpinf1, Sparc, Tmsb10, Ubc	15

	**Decreased**		0
	**Stable**	Anti-dectin-1 15E2 heavy chain, Anti-dectin-1 15E2 light chain, Hist1h1b, Hist1h1d, Tf	5
	**Variable**	Agt, Apoa1, Apoe, Hbb-b2, Hist1h1e, Hist2h4, Hpx, Saa3, Ybx1	9
**CIS+LH**	**Appeared**	Abracl, Adam10, Adam12, Adamts1, Adamts2, Akr1a1, Apoe, Atox1, Atp5a1, Bmp1, Ccdc80, Cdh5, Cfl1, Clu, Col15a1, Col1a2f, Col3a1, Col4a1, Col4a2, Col5a1, Col5a2, Col6a1, Colec12, Cpe, Cstb, Ctla2a, Ctsb, Ctsl, Dbi, Ddt, Eln, Eno1, Fn1, Fstl1, Grn, Gsn, Hist2h4, Hnrnpk, Hspe1, Hspg2, Ifnar2, Igf1, Igfbp2, Igfbp3, Igfbp4, Il4r, Inha, Itih5, Ldha, Ldhb, Lox, Loxl1, Ltbp1, Lum, Lyz2, Mmp14, Mmp2, Nedd8, Nid2, Nme1, Nucb1, Pabpc1, Pebp1, Ppic, Prdx1, Prdx2, Prdx5, Prdx6, Prnpb, Prss23, Psap, Pxdn, Qsox1, Rbmx, Rnase4, Rpl6, Rplp1, Rplp2, Rps21, Rps28, S100a11, Tuba1c, Ube2n, Uchl1, Vim, Zp2, Zp3	87
	**Disappeared**	Isoc1	1
	**Accumulated**	B2m, Calm1, Col18a1, Col1a1, Col1a2, Cst3, Dag1, Htra1, Igfbp5, Itm2b, Lgals1, Mif, Ppia, Prss35, Serpinf1, Sod1, Sparc, Tagln2, Tmsb10, Tpi1, Ubc	20
	**Decreased**	Anti-dectin-1 15E2 heavy chain, Hba	2
	**Stable**	Actg1, Anti-dectin-1 15E2 light chain, Hbb-b2, Hist1h1b, Hist1h1d, Hist1h1e, Igf2, Tf, Ybx1	9
	**Variable**	Apoa1, Col6a3, Eef1a1, Gapdh, Hbat1, Hpx, Igfbp7, Pgam1, Pkm, Saa3, Tmsb4x	11

Table S3. Shared proteins between indicated groups. Proteins shared are listed

<table>
<tr><td rowspan="3">Shared by CTRL, LH, CIS and CIS+LH</td><td>2h</td><td>Col1a1, Serpinf1, Dag1, Lgals1, Gapdh, Hpx, Actg1, B2m, Col1a2, Mif, Ppia, Cst3, Itm2b, Sparc, Hist1h1d, Tf, Anti-dectin-1 15E2 heavy chain, Anti-dectin-1 15E2 light chain</td><td>18</td></tr>
<tr><td>12h</td><td>Fn1, Tpi1, Col4a1, Itih5, Adamts2, Hpx, Col18a1, Psap, Col4a2, Serpinf1, Igfbp5, Cfl1, Clu, Fstl1, Igfbp7, Tagln2, Vim, Lox, Loxl1, Lum, Igfbp2, Zp2, Hist1h1b, Anti-dectin-1 15E2 heavy chain, Calm1, Eef1a1, Igf2, Dbi, Gapdh, Dag1, Sod1, Hspe1, Zp3, Igfbp4, Col3a1, Actg1, Prss35, Eln, Ppia, Mif, Anti-dectin-1 15E2 light chain, B2m, Lgals1, Hist1h1e, Col1a1, Tmsb10, Sparc, Lyz2, Col1a2, Saa3, Hist1h1d, Itm2b,, Cst3, Tf, Tmsb4x</td><td>55</td></tr>
<tr><td>24h</td><td>Actg1, Adamts1, Adamts2, Apoe, Atox1, B2m, Bmp1, Calm1, Cdh5, Clu, Col15a1, Col18a1, Col1a1, Col1a2, Col1a2f, Col3a1, Col4a1, Col4a2, Col5a2, Col6a1, Col6a3, Colec12, Cpe, Cst3, Ctla2a, Ctsb, Dag1, Dbi, Ddt, Eef1a1, Eno1, Eln, Fn1, Fstl1, Gapdh, Grn, Hbat1, Hbb-b2, Hist1h1b, Hist1h1d, Hist1h1e, Hpx, Hspe1, Hspg2, Igf2, Igfbp2, Igfbp3, Igfbp4, Igfbp5, Igfbp7, Inha, Itih5, Itm2b, Ldhb, Lgals1, Lox, Loxl1, Ltbp1, Lum, Lyz2, Mif, Mmp14, Mmp2, Nedd8, Nid2, Nme1, Nucb1, Pabpc1, Pebp1, Pgam1, Ppia, Ppic, Prdx1, Prdx6, Prnpb, Prss35, Psap, Pxdn, Qsox1, Rnase4, Rplp2, Rps28, Saa3, Serpinf1, Sod1, Sparc, Tagln2, Tf, Tmsb10, Tmsb4x, Tpi1, Ube2n, Uchl1, Vim, Ybx1, Zp2, Zp3, Anti-dectin-1 15E2 heavy chain, Anti-dectin-1 15E2 light chain</td><td>99</td></tr>
<tr><td rowspan="3">Shared by CTRL, LH and CIS</td><td>2h</td><td>Actg1, B2m, Col1a1, Col1a2, Cst3, Dag1, Gapdh, Hist1h1d,, Hpx, Itm2b, Lgals1, Mif, Ppia, Serpinf1, Sparc, Tf, Anti-dectin-1 15E2 heavy chain, Anti-dectin-1 15E2 light chain</td><td>18</td></tr>
<tr><td>12h</td><td>Actg1, Adamts2, B2m, Calm1, Cfl1, Clu, Col18a1, Col1a1, Col1a2, Col3a1, Col4a1, Col4a2, Cst3, Dag1, Dbi, Eef1a1, Eln, Fn1, Fstl1, Gapdh, Hist1h1b, Hist1h1d, Hist1h1e, Hpx, Hspe1, Hspg2, Igf2, Igfbp2, Igfbp3, Igfbp4, Igfbp5, Igfbp7, Itih5, Itm2b, Lgals1, Lox, Loxl1, Lum, Lyz2, Mif, Ppia, Prss35, Psap, Saa3, Serpinf1, Sod1, Sparc, Tagln2, Tf, Tmsb10, Tmsb4x, Tpi1, Vim, Zp2, Zp3, Anti-dectin-1 15E2 heavy chain, Anti-dectin-1 15E2 light chain</td><td>57</td></tr>
<tr><td>24h</td><td>Actg1, Adamts1, Adamts2, Apoe, Atox1, B2m, Bmp1, Calm1, Cdh5, Clu, Col15a1, Col18a1, Col1a1, Col1a2, Col1a2f, Col3a1, Col4a1, Col4a2, Col5a2, Col6a1, Col6a3, Colec12, Cpe, Cst3, Ctgf, Ctla2a, Ctsb, Dag1, Dbi, Ddt, Dkk3, Eef1a1, Eno1, Eln, Fbn1, Fn1, Fstl1, Gapdh, Grn, Hbat1, Hbb-b2, Hist1h1b, Hist1h1d, Hist1h1e, Hpx, Hspe1, Hspg2, Igf2, Igfbp2, Igfbp3, Igfbp4, Igfbp5, Igfbp7, Inha, Itih5, Itm2b, Ldhb, Lgals1, Lox, Loxl1, Ltbp1, Lum, Lyz2, Mif, Mmp14, Mmp2, Nedd8, Nid2, Nme1, Nucb1, Pabpc1, Pebp1, Pgam1, Ppia, Ppic, Prdx1, Prdx6, Prnpb, Prss35, Psap, Pxdn, Qsox1, Rnase4, Rplp2, Rps28, Saa3, Serpinf1, Serping1, Sod1, Sparc, Tagln2, Tf, Tmsb10, Tmsb4x, Tpi1, Ube2n, Uchl1, Vim, Ybx1, Zp2, Zp3, Anti-dectin-1 15E2 heavy chain, Anti-dectin-1 15E2 light chain</td><td>103</td></tr>
<tr><td rowspan="3">Shared by CTRL, CIS and CIS+LH</td><td>2h</td><td>Actg1, B2m, Col1a1, Col1a2, Cst3, Dag1, Gapdh, Hist1h1d, Hist1h1e, Hpx, Itm2b, Lgals1, Mif, Ppia, Serpinf1, Sparc, Tf, Anti-dectin-1 15E2 heavy chain, Anti-dectin-1 15E2 light chain</td><td>19</td></tr>
<tr><td>12h</td><td>Actg1, Adamts2, B2m, Calm1, Cfl1, Clu, Col18a1, Col1a1, Col1a2, Col3a1, Col4a1, Col4a2, Col6a3, Cst3, Dag1, Dbi, Eef1a1, Eln, Fn1, Fstl1, Gapdh, Hist1h1b, Hist1h1d, Hist1h1e, Hpx, Hspe1, Igf1, Igf2, Igfbp2, Igfbp4, Igfbp5, Igfbp7, Itih5, Itm2b, Lgals1, Lox, Loxl1, Lum, Lyz2, Mif, Ppia, Prss35, Psap, Saa3, Serpinf1, Sod1, Sparc, Tagln2, Tf, Tmsb10, Tmsb4x, Tpi1, Vim, Zp2, Zp3, Anti-dectin-1 15E2 heavy chain, Anti-dectin-1 15E2 light chain</td><td>57</td></tr>
<tr><td>24h</td><td>Actg1, Adam10, Adam12, Adamts1, Adamts2, Alyref, Apoe, Atox1, B2m, Bmp1, Calm1, Cdh5, Clu, Col15a1, Col18a1, Col1a1, Col1a2, Col1a2f, Col3a1, Col4a1, Col4a2, Col5a2, Col6a1, Col6a3, Colec12, Cpe, Cst3, Cstb, Ctla2a, Ctsb, Ctsl, Dag1, Dbi, Ddt, Eef1a1, Eno1, Eln, Fn1, Fstl1, Gapdh, Grn, Hbat1, Hbb-b2, Hist1h1b, Hist1h1d, Hist1h1e, Hist2h4, Hpx, Hspe1, Hspg2, Ifnar2, Igf2, Igfbp2, Igfbp3, Igfbp4, Igfbp5, Igfbp7, Inha, Itih5, Itm2b, Ldhb, Lgals1, Lox, Loxl1, Ltbp1, Lum, Lyz2, Mif, Mmp14, Mmp2, Nedd8, Nid2, Nme1, Nucb1, Pabpc1, Pebp1, Pgam1,</td><td>110</td></tr>
</table>

		Ppia, Ppic, Prdx1, Prdx5, Prdx6, Prnpb, Prss35, Psap, Pxdn, Qsox1, Rbmx, Rnase4, Rplp1, Rplp2, Rps28, S100a11, Saa3, Serpinf1, Sod1, Sparc, Tagln2, Tf, Tmsb10, Tmsb4x, Tpi1, Ube2n, Uchl1, Vim, Ybx1, Zp2, Zp3, Anti-dectin-1 15E2 heavy chain, Anti-dectin-1 15E2 light chain	
**Shared by CTRL, LH and CIS+LH**	**2h**	Actg1, B2m, Col1a1, Col1a2, Cst3, Dag1, Gapdh, Hbat1, Hist1h1d, Hpx, Itm2b, Lgals1, Mif, Ppia, Serpinf1, Sparc, Tf, Tmsb4x, Anti-dectin-1 15E2 heavy chain, Anti-dectin-1 15E2 light chain	20
	**12h**	Actg1, Adamts2, B2m, Calm1, Cfl1, Clu, Col18a1, Col1a1, Col1a2, Col3a1, Col4a1, Col4a2, Cst3, Dag1, Dbi, Eef1a1, Eln, Fn1, Fstl1, Gapdh, Hist1h1b, Hist1h1d, Hist1h1e, Hpx, Hspe1, Igf2, Igfbp2, Igfbp4, Igfbp5, Igfbp7, Itih5, Itm2b, Lgals1, Lox, Loxl1, Lum, Lyz2, Mif, Mmp2, Ppia, Prss35, Psap, Rps28, Saa3, Serpinf1, Sod1, Sparc, Tagln2, Tf, Tmsb10, Tmsb4x, Tpi1, Vim, Zp2, Zp3, Anti-dectin-1 15E2 heavy chain, Anti-dectin-1 15E2 light chain	57
	**24h**	Actg1, Adamts1, Adamts2, Apoe, Atox1, Atp5a1, B2m, Bmp1, Calm1, Cdh5, Clu, Col15a1, Col18a1, Col1a1, Col1a2, Col1a2f, Col3a1, Col4a1, Col4a2, Col5a2, Col6a1, Col6a3, Colec12, Cpe, Cst3, Ctla2a, Ctsb, Dag1, Dbi, Ddt, Eef1a1, Eno1, Eln, Fn1, Fstl1, Gapdh, Grn, Gsn, Hbat1, Hbb-b2, Hist1h1b, Hist1h1d, Hist1h1e, Hpx, Hspe1, Hspg2, Htra1, Igf2, Igfbp2, Igfbp3, Igfbp4, Igfbp5, Igfbp7, Inha, Itih5, Itm2b, Ldha, Ldhb, Lgals1, Lox, Loxl1, Ltbp1, Lum, Lyz2, Mif, Mmp14, Mmp2, Nedd8, Nid2, Nme1, Nucb1, Pabpc1, Pebp1, Pgam1, Ppia, Ppic, Prdx1, Prdx2, Prdx6, Prnpb, Prss23, Prss35, Psap, Pxdn, Qsox1, Rnase4, Rplp2, Rps28, Saa3, Serpinf1, Sod1, Sparc, Tagln2, Tf, Tmsb10, Tmsb4x, Tpi1, Ube2n, Uchl1, Vim, Ybx1, Zp2, Zp3, Anti-dectin-1 15E2 heavy chain, Anti-dectin-1 15E2 light chain	105
